# Does Premedication with Mucolytic Agents Improve Mucosal Visualization during Oesophagogastroduodenoscopy: A Systematic Review and Meta-Analysis

**DOI:** 10.1155/2021/1570121

**Published:** 2021-01-22

**Authors:** Eoghan Burke, Patricia Harkins, Frank Moriarty, Ibrahim Ahmed

**Affiliations:** ^1^Our Lady of Lourdes Hospital, Drogheda, Co Louth, Ireland; ^2^St James's Hospital, Dublin, Ireland; ^3^HRB Centre for Primary Care Research, RCSI Department of General Practice, RCSI, 123 St Stephens Green, Dublin, Ireland

## Abstract

**Introduction:**

Gastric Cancer (GC) is the fourth most common malignancy worldwide and the second leading cause of cancer-related mortality for both sexes. The gold standard for diagnosing GC is oesophagogastroduodenoscopy (OGD). Excess mucus on the gastric mucosa impairs the detection of early GC.

**Aim:**

To synthesize available evidence of the effect of premedication with a mucolytic agent among adults undergoing elective nontherapeutic OGD, compared to placebo or other mucolytic agents, on mucosal visibility during OGD.

**Methods:**

A systematic review was conducted. PubMed, EMBASE, CINAHL, Cochrane central register of controlled trials (CENTRAL), and Web of Science were searched for relevant studies. A random-effects meta-analysis was performed to determine the mean difference in total mucosal visibility score (TMVS) between the pooled mucolytic agents and control. Subgroup analyses were performed to determine the mean TMVS difference for simethicone versus control and the impact of different timings and doses of mucolytic premedication.

**Results:**

13 studies, involving 11,086 patients, including 6178 females (55.7%), with a mean age of 53.4 were identified and 6 of these were brought forward to meta-analysis. This revealed a mean difference of −2.69 (95% CI −3.5, −1.88) in total mucosal visibility scores (TMVS) between the pooled mucolytic agents and control. For simethicone, the mean difference was −2.68 (95% CI −4.94, −0.43). A simethicone dose of 133 mg was most effective with a mean difference of −4.22 (95% CI −5.11, −3.33). Assessing timing of administration across all mucolytic agents revealed a mean difference for the >20 minutes group of −3.68 (95% CI −4.77, −2.59). No adverse events were reported in any included trials.

**Conclusions:**

Regular use of premedication with mucolytic agents prior to routine OGD is associated with improved TMVS with no reported adverse events.

## 1. Introduction

Gastric Cancer (GC) is the fourth most common malignancy worldwide and the second leading cause of cancer-related mortality for both sexes. It is estimated that approximately 22,220 patients in the USA will be diagnosed with gastric cancer annually [1]. The highest incidence rates globally are found in the far east, with South Korea being identified as having the highest rate of gastric cancer in 2018 with an age standardized rate of 39.6 per 100,0002. Japan ranks third in terms of incidence rates for GC with an age standardized rate of 27.5 per 100,000 [2]. Globally it is also important to note that whilst the overall incidence rates of GC are declining, there is an upward trend in younger patients [3] and the prevalence of GC is expected to increase over the coming decade secondary to the growing population [4].

Advanced GC has a poor prognosis with 5-year survival for stage 4 disease of 5.2% [5]. Therefore, it is imperative that it be identified at as early a stage as possible. The gold standard for diagnosing GC is oesophagogastroduodenoscopy (OGD). OGD is a dynamic test which is technically challenging and can be further hampered by excess mucus obscuring a clear view of the gastric mucosa. This has been acknowledged by the Japanese Society of Gastroenterology which has had guidelines on quality assurance for OGD for many years [5]. These guidelines support the routine use of pharmacological agents to aid the visualization of the gastric mucosa during OGD including the routine use of mucolytic agents to remove excess mucus from the mucosa. The issue of quality standards for OGD in the UK and Ireland has been highlighted recently. In September 2017, the British Society Of Gastroenterology, in association with the Association of Upper Gastrointestinal Surgeons of Great Britain and Ireland, published their first position paper on the topic. The position paper makes 38 recommendations for quality assurance during OGD including “adequate mucosal visualization should be achieved by a combination of adequate air insufflation, aspiration and the use of mucosal cleansing techniques” [6].

The techniques advocated include the use of water flushes and whilst the recommendation does briefly discuss the use of mucolytic agents, the grade of evidence for their routine use was graded as moderate and so their routine use is not advocated under this current guideline.

### 1.1. Study Aim and Objectives

The aim of this study is to synthesize available evidence of the effect of premedication with a mucolytic agent among adults undergoing elective nontherapeutic OGD, compared to placebo or other mucolytic agents, on mucosal visibility during OGD.

The specific objectives of this systematic review and meta-analysis include the following:

- To assess the effect of premedication with mucolytics on mucosal visibility during OGD
- To assess the effect of different mucolytics, doses, and administration timing on mucosal visibility
- To assess the incidence of adverse effects of mucolytic agents

The PICO model was used to devise the search criteria, defined in detail in Table 1.

## 2. Methods

### 2.1. Study Design

This study was a systematic review and meta-analysis of published randomized controlled trials. Reporting of this systematic review is in accordance with the Preferred Reporting Items for Systematic Reviews and Meta-Analyses (PRISMA) statement [7] and was registered with the PROSPERO register (registration number: CRD42019133964).

### 2.2. Inclusion and Exclusion Criteria

Inclusion criteria were as follows:

- Randomized controlled clinical trials
- Adult patients (>18 years old)
- Elective OGD
- Intervention: premedication with any mucolytic agent including pronase, simethicone, dimethicone, and N-acetylcysteine
- Comparator: placebo, water, or another mucolytic agent
- Outcome: improved mucosal visibility as determined by a defined scoring system, e.g., the Total Mucosal Visibility Score (TMVS) [8]

Exclusion Criteria:

- Paediatric participants
- Emergency OGDs, Therapeutic OGDs
- Articles available only as abstract.

### 2.3. Search Strategy

A detailed search strategy was developed in consultation with an information specialist (P. Murphy). Key words and MeSH terms relating to OGD and mucolytic agents were used to develop the search string: (OGD OR EGD OR gastroscopy OR Oesophagogastroduodenoscopy OR Esophagogastroduodenoscopy) AND (mucolytic OR “N-Acetylcysteine” OR N-Acetyl-L-cysteine OR “N Acetyl L cysteine” OR N-Acetylcysteine OR pronase OR “Protease XIV” OR “XIV Protease” OR “Pronase P” OR “Pronase E” OR dimethicone OR “dimethyl polysiloxane” OR simethicone OR “Phazyme 125”). This search string was applied to the bibliographic databases: PubMed, EMBASE, CINAHL, Cochrane central register of controlled trials (CENTRAL), and Web of Science. This combination of bibliographic databases was chosen based on the findings by Bramer et al. [9] on the optimum database combinations to be searched for a systematic review. There was no language restriction imposed on the search. All databases were searched from inception.

### 2.4. Study Selection

After duplicates were removed, all of the identified studies' titles and abstracts were independently screened by two of the authors (E Burke and P Harkins). Abstracts meeting the previously described inclusion criteria were selected. If there was any conflict about a study's inclusion, this was resolved by a third author (F Moriarty). The resulting studies were then reviewed in full and eligibility for inclusion in qualitative and quantitative analysis determined. Any conflict pertaining to a study's eligibility was resolved with consensus. During full article review, hand searching of references to identify any studies not identified in the original search was conducted. Similarly, a citation search using Google Scholar on all eligible articles was completed again to ensure no further studies were omitted [9].

### 2.5. Data Extraction

Two of the authors (E Burke and P Harkins) independently extracted data from the selected studies using a predetermined data extraction form. Data extracted included study authors, year of publication, journal of publication, study design, participant demographics, intervention details, control details, outcome measure (a form of visibility score used), anatomical sites assessed during the OGD, the timing of premedication administration, and results including adverse event data.

### 2.6. Risk of Bias Assessment

The quality and risk of bias in each study were assessed independently by two authors (E Burke and P Harkins) using the Cochrane collaborations risk of bias tool [10]. The susceptibility was rated as either low risk, high risk, or unclear risk. The results were depicted graphically using RevMan software.

### 2.7. Summary Measures and Synthesis of Results

We conducted a qualitative assessment (systematic review) of all eligible studies. Studies reporting the primary outcome of mucosal visibility using the most common TMVS were then synthesized quantitatively using meta-analysis.

The most commonly used TMVS is derived by McNally et al. (please see the appendix (Figure 9)). The McNally score involves assessing the quality of mucosal visibility at 4 anatomical sites in the upper GI tract, often 4 sites within the stomach, e.g., Antrum, Lower Body, Upper Body, and fundus. The quality of mucosal visibility at each site then scores from 1 to 4. A score of 1 denotes no adherent mucous on the gastric mucosa, a score of 2 denotes a small amount of mucous on the gastric mucosa but not obscuring vision, a score of 3 denotes a large amount of mucous on the gastric mucosa requiring washing with less than 50 ml of water to improve visibility and finally a score of 4 is assigned if a volume of greater than 50 ml of water is required to improve visibility. The sum score from each of the 4 sites is added to yield the total Mucosal Visibility Score. The score thus runs from a minimum of 4 to a maximum of 16.

Studies comparing either a single mucolytic agent or a combination of mucolytic agents versus control were pooled together. The mean and standard deviation of the TMVS and number of participants in each group were then extracted to facilitate a pairwise meta-analysis to determine the mean difference. Mean and standard deviation were used as the TMVS is a continuous outcome measure, where the TMVS was reported as a median and interquartile range. The technique developed by Wan et al. [11] was used to convert the TMVS to a mean and standard deviation (SD), where confidence intervals (CI) were reported as opposed to SD. The technique supported by the Cochrane Collaboration was used to convert the CI to SD.

If at least two studies comparing a single mucolytic agent versus control were available, these were then compared. Similarly, if at least two studies comparing the same combination of mucolytic agents versus control were available, these were compared. Care was taken to avoid making a unit of analysis error in cases of studies with multiple intervention or control arms.

Statistical heterogeneity amongst the studies was calculated using *I*^2^. A *P*-value of less than 0.05 was considered significant where appropriate.

The effect of timing of premedication administration on mean difference in TMVS between the pooled mucolytic agents versus control was assessed and graphed. If at least two studies compared the same mucolytic agent versus control were available, the effect of mucolytic dose on mean difference in TMVS between the mucolytic agent and control was assessed and graphed.

The statistical analysis of the data will be conducted using the Cochrane Collaboration guidelines including the use of RevMan 5.3® statistical software.

## 3. Results

### 3.1. Study Selection

The number of articles found via searching the bibliographic databases PubMed, EMBASE, CINAHL, Cochrane Central Register of Controlled Trials, and Web of Science was 415. A further 10 articles were found by searching the clinical trial registries including Clinicaltrials.gov, Japanese Medical Association Clinical Trials Registry, and the EU clinical trials register. No further relevant studies were identified by hand searching of references or via citation searching on Google Scholar. Following the removal of duplicates, the number of original articles to screen was 366. Screening of the title and abstract of these articles was performed independently by E Burke and P Harkins. Articles meeting criteria for further evaluation of full text numbered 18. Of these 18 studies, 5 were excluded as they had a different primary outcome measure (namely, the effect of premedication with the mucolytic agent on the volume of flushes needed during OGD). Thus, 13 studies were included in the final review for narrative synthesis.

**Figure 1:**
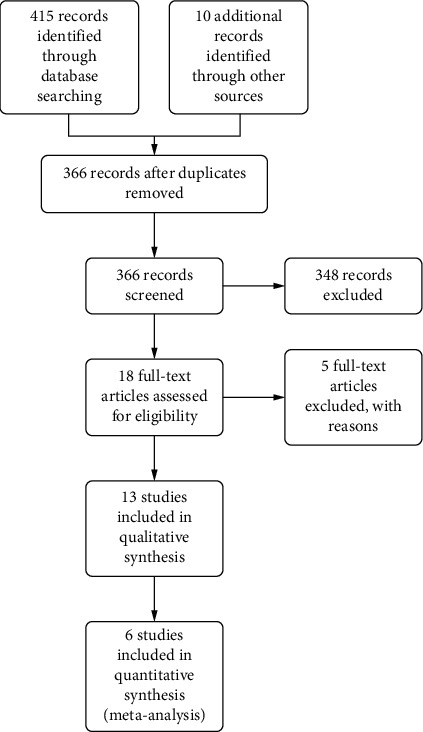
Prisma flowchart, preferred reporting items for systematic reviews and meta-analyses. This depicts the selection of studies for meta-analysis.

Of these 13 studies, 6 were deemed suitable to include in the meta-analysis. They were deemed suitable as they all used the same TMVS system and they all contained a suitable control group to facilitate a pairwise meta-analysis. All 13 studies were compared qualitatively. Please see Figure 1 which depicts the relevant PRISMA Flowchart describing the process of including studies within this review.

### 3.2. Study Characteristics

The characteristics of the studies included are detailed in Table 2. A total of 13 studies were included in this systematic review [8, 12–23]. The number of patients included in the studies ranged from a low of 54 to a high of 7143. The total number of patients in all 13 studies was 11,086 including 6178 females (55.7%) and 4908 males (44.3%). The mean age of participants in the 13 studies was 53.4.

9 of the included studies employed the most commonly used McNally TMVS. One study used a dichotomous scoring system assigning either excellent or not excellent to describe the quality of mucosal visibility. The remaining 3 studies used a modified version of the McNally scoring system. This system grades each area from 1 to 3 in terms of quality of mucosal visibility, thus yielding a minimum score of 3 and a maximum score of 12.

There was similarly significant heterogeneity between the studies in relation to the anatomical sites to which the scoring system was applied. 8 of the studies applied the TMVS to the recommended 4 anatomical sites. 2 studies applied it to 5 anatomical sites, and 1 study applied the score to each of 3, 6, and 7 sites.

Similarly, there was significant heterogeneity between included studies in relation to the timing of premedication. The timings ranged from 5 to 30 mins with a mean of 17.7 mins.

All of the most commonly used mucolytic agents including dimethicone, simethicone, N-Acetylcysteine, and pronase were used within the 13 studies. There was significant heterogeneity within the studies in relation to doses and combinations of mucolytic agents used. 6 of the included studies did not have a control group.

All included studies reported no adverse events and all studies reported, using 1 of the 3 aforementioned scoring systems, improved mucosal visibility when premedication with mucolytic agents are used.

### 3.3. Risk of Bias Assessment

**Figure 2:**
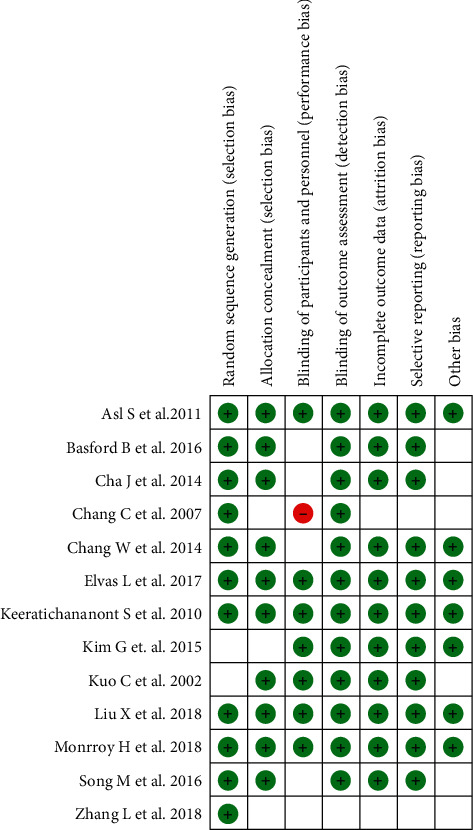
Risk of bias summary: review authors' judgments about each risk of bias item for each included study. The green circle indicates low risk of bias, the red circle indicates high risk, and a blank box indicates unclear risk.

**Figure 3:**
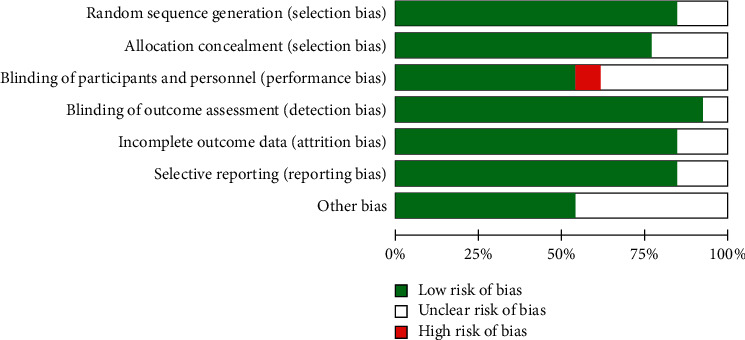
Risk of bias graph: review authors' judgments about each risk of bias item presented as percentages across all included studies.

Study quality in terms of risk of bias was assessed independently by E Burke and P Harkins using the Cochrane Collaboration risk of bias tool. Each study was assessed in terms of susceptibility in relation to selection bias, performance bias, detection bias, attrition bias, and reporting bias. The results are outlined in Figures 2 and 3. The risk of selection bias (with evidence of random sequence generation) was low in 11 of the 13 studies and unclear in 2. The mechanism of random sequence generation was reported in only 6 of 11 studies which reported using it. The risk of selection bias in terms of allocation concealment was low in 10 of the 13 studies and unclear in 3. The risk of performance bias was low in 7 of the 13 studies, high in 1 study, and unclear in 5 studies. The risk of detection bias was low in 12 studies and unclear in 1 study. The risk of attrition bias was low in 11 of the studies and unclear in 2 of the studies. The risk of reporting bias was low in 11 of the studies and unclear in 2. Overall, the studies appeared to be most resistant to detection bias, with 12 of the 13 studies having clear evidence of blinding of outcome assessment with evidence of an independent investigator reporting the TMVS. The studies appear to be most susceptible to performance bias in terms of blinding of participants and endoscopists, with 7 of the studies having clear evidence of blinding, 5 studies having unclear evidence of blinding, and 1 study by Chang et al. having a high risk.

### 3.4. Synthesis of Results for Meta-Analysis

As previously described, 6 of the 13 studies were deemed suitable for a pairwise meta-analysis to determine the effect of any mucolytic agent compared to control on TMVS. Each of these studies employed the McNally scoring system and had a suitable control group. The mean and standard deviation for the TMVS for mucolytic agents in each study was extracted or calculated. In studies with multiple intervention arms using different combinations of mucolytic agents at different doses, these arms were pooled together. The TMVS score for the study by Monrroy et al. was reported as a median with interquartile ranges and so mean and standard deviation was calculated using the technique developed by Wan et al. The TMVS in the study by Basford et al. was reported as mean with confidence intervals and so standard deviation was determined using the technique endorsed by the Cochrane Collaboration [24].

**Figure 4:**
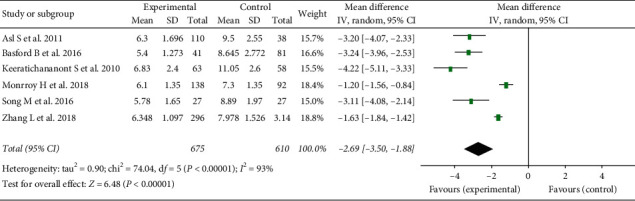
Forest plot of Total Mucosal Visibility Score (McNally) for pooled mucolytic agent vs. control.

A random-effects meta-analysis was then performed to determine the mean difference in TMVS between the pooled mucolytic agents and control. The pooled mucolytic agents included data for simethicone, pronase, and N-Acetylcysteine. Results were graphed in a forest plot (Figure 4). This revealed a mean difference of −2.69 with a 95% CI of between −3.5 and −1.88. The Z statistic for the overall effect size was 6.48 and was statistically significant with a *P*-value of <0.00001. There was significant heterogeneity between included studies, as evidenced by the *I*^2^ value of 93%.

#### 3.4.1. Simethicone Studies

**Figure 5:**
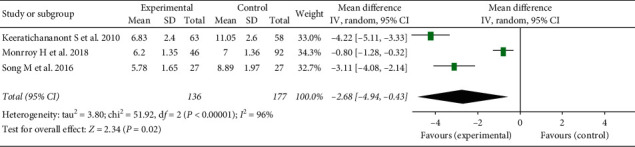
Forest plot of the Total Mucosal Visibility Score (McNally) between simethicone premedication agent and control.

By evaluating the study characteristics in terms of the intervention arms of each study, it was determined that only simethicone was examined as an individual agent in at least two studies (namely, Keeratichananont et al. 2010, Monrroy et al. 2018, and Song et al. 2016). Thus, a subgroup analysis was performed (Figure 5). This revealed a mean difference of −2.68 with a 95% confidence interval of −4.94 to −0.43. The Z statistic for the overall effect size was 2.34 and was statistically significant with a *P*-value of <0.02. There was significant heterogeneity, as evidenced by the *I*^2^ of 96%.

#### 3.4.2. Combined Mucolytics

**Figure 6:**
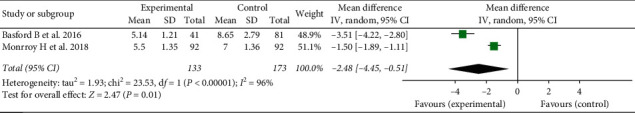
Forest plot of Total Mucosal Visibility Score (McNally) between combination premedication agent (Simethicone + N- Acetylcysteine) and control.

By evaluating the study characteristics, it was determined that only the combination of simethicone and N-Acetylcysteine versus control was assessed in at least two studies, namely Basford et al. 2016 and Monrroy et al. 2018. Thus, a subgroup analysis was performed (Figure 6.). This revealed a mean difference of −2.48 with a 95% confidence interval of −4.45 to −0.51. The Z statistics for the overall effect size were 2.47 and were statistically significant, with a *P*-value of <0.01. There was significant heterogeneity as evidenced by the *I*^2^ of 96%.

#### 3.4.3. Simethicone Dose

**Figure 7:**
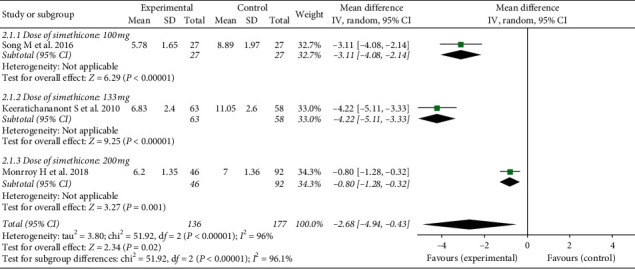
Forest plot of subgroup analysis of the effect of the dose of simethicone on the mean difference in TMVS between simethicone and control agents.

The effect of dose of simethicone on mean difference in TMVS between simethicone premedication and control was then assessed by conducting a subgroup analysis on the studies which used simethicone as a single premedication compared to control. These studies included Song et al. 2016 using 100 mg simethicone, Keeratichananont et al. 2010 using 133 mg of simethicone, and Monrroy et al. 2018 using 200 mg simethicone. The results were graphed on a forest plot (Figure 7).

The mean difference for the 100 mg simethicone subgroup was −3.11 with a 95% confidence interval of between −4.08 and −2.14. The overall effect Z test was 6.29. This was statistically significant, as evidenced by the *P* value of <0.00001. The mean difference for the 133 mg simethicone subgroup was −4.22 with a 95% confidence interval of between −5.11 and −3.33. The overall effect Z test was 9.25. This was statistically significant with a *P* value of <0.00001. The mean difference for the 200 mg of simethicone subgroup was −0.8 with a 95% confidence interval of between −1.28 and −0.32. The overall effect Z test was 3.27. This was found to be statistically significant as evidenced by the *P* value = 0.001.

#### 3.4.4. Timing of Mucolytic Administration

The effect of timing of premedication administration was assessed by conducting a subgroup analysis. Based on the study characteristics of the 6 RCTs, they were grouped into two subgroups based on the timing of premedication. Timings were categorized as >20 minutes to denote the two studies whose timing was greater than 20 minutes (Keeratichananont et al. 2010 at 15–30 mins and Song et al. 2016 at 30 mins), and </ = 20 minutes for the 4 RCTs whose premedication was administered at less than or equal to 20 minutes (Asl et al. 2011 at 20 mins, Basford et al. 2016 at 5–10 mins, Monrroy et al. 2018 at 20 mins, and Zhang et al. 2018 at 20 mins).

**Figure 8:**
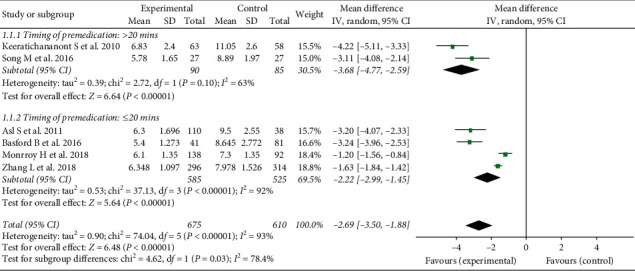
Forest plot of subgroup analysis of the effect of timing of administration of premedication on mean difference in TMVS between pooled mucolytic agents and control.

**Figure 9:**
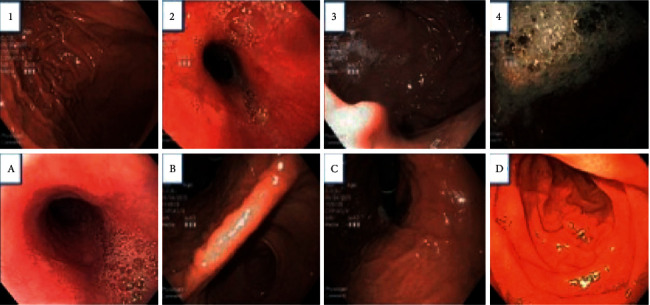
Endoscopic scoring system. Score of 1: No bubbles; Score of 2: Minimal bubbles which the endoscopist must actively look for; Score of 3: Foam is obviously present but not severe; Score of 4: Severe foam obscuring vision; Area (A): Esophagus; Area (B): The antrum and angularis of the stomach; Area (C): The body and fundus of the stomach; Area (D): Duodenum. TMVS is the sum of the scores of areas A, B, C, and D added together. TMVS: total mucosal visibility score.

The results were graphed on a forest plot (Figure 8). This revealed a mean difference for the >20 minutes group of −3.68 with a 95% confidence interval of −4.77 to −2.59. There was significant heterogeneity between the studies, as evidenced by the *I*^2^ of 63%. The overall effect Z statistic was 6.64. This was statistically significant, with a *P* value of <0.00001.

The mean difference for the less than or equal to 20 minutes group was −2.69 with a 95% confidence interval of between −2.99 and −1.45. There was significant heterogeneity between the included studies, as evidenced by the *I*^2^ of 93%. The overall effect Z statistic was 5.64. This was statistically significant with a *P* value of <0.00001.

## 4. Discussion

OGD is the gold standard for diagnosing GC. It is well documented that impaired mucosal visibility secondary to adherent mucus is a potential cause for incomplete OGD and thus missed early GC. The term “interval GC” is used to describe a GC diagnosed in a patient who underwent OGD within the preceding 3 years, with the implication that early signs of neoplasia were missed at that time. The precise rate of interval GCs is not well reported in either the UK or Ireland. A recent study conducted by Woodland et al. in the Royal United Hospital in Bath reported an interval cancer rate of 7.3% [25]. There are multiple factors that contribute to incomplete mucosal visualization during OGD as previously described. We conducted this systematic review and meta-analysis with the aim of providing evidence for or against the use of mucolytic agents as premedication prior to diagnostic OGD. We also wanted to provide guidance on dosage, the timing of administration, and side effects if any were noted.

Our extensive literature search identified 13 relevant randomized controlled trials which examined the effect of a mucolytic agent or combination of agents on mucosal visibility when administered as a premedication. Our narrative review of these studies revealed significant heterogeneity amongst the studies. The use of multiple different scoring systems for assessing mucosal visibility is an issue that was previously described by Sajid et al. [25]. This limits our ability to accurately compare the effects between studies. Similarly, within the 13 studies, there was a significant variation in the anatomical sites within the upper gastrointestinal tract to which the scoring system was applied. Variation in outcome measurement across studies is common and presents challenges for evidence synthesis.

After assessing the characteristics of the included studies, we determined that 6 of them would be appropriate to take forward to conduct a quantitative assessment on. These studies included 1285 patients, including 749 females (58%) and 536 males (42%), with a mean age of 52.5. These 6 studies were deemed appropriate as they used the same TMVS system and applied it to the same anatomical locations. They also all used a control group that facilitated pairwise meta-analysis. Within these 6 studies, all the commonly used mucolytic agents were used either alone or in various combinations. This included dimethicone which is most commonly used in the USA, pronase which is most commonly used in Japan, and simethicone which is available in Ireland and the UK as Infacol. To our knowledge, this was the first time the effect of these agents was pooled together to assess the effect on mucosal visibility. The forest plot (Figure 4) revealed a mean difference of −2.69 with a 95% confidence interval of between −3.5 and −1.88 in favor of mucolytic agents (in general) being superior to control in improving mucosal visibility. If we couple this with the absence of adverse events reported in any of the 13 trials, which included a total of 11,086 patients, we feel the routine use of a mucolytic agent during diagnostic OGD is justified and should be endorsed in future guidelines.

We then attempted to determine which mucolytic agent was superior; however, due to the heterogeneity of the studies, we were only able to assess the effect of simethicone alone and in combination with N-Acetylcysteine. The subgroup analysis of the 3 studies using simethicone alone compared to a control (namely, Keeratichananont et al. 2010, Monrroy et al. 2018, and Song et al. 2016) revealed a mean difference of −2.68 with a 95% confidence interval of −4.94 to −0.43. This was similar to the results obtained by Sajid et al. in their systematic review of the use of simethicone in improving mucosal visibility (standardized mean difference −2.83 with 95% confidence interval of between −4.38 and −1.27).

The mean effect of simethicone when used alone was −2.68 compared to the mean effect of the pooled mucolytic agents of −2.69. Thus potentially, simethicone may be as good as or better than any of the other agents included in the pooled meta-analysis. Although there was a notable difference in effect size between the pooled mucolytic agent mean difference of 6.48 compared to the effect size of 2.34 for simethicone alone. This could only fully be elucidated further using a network meta-analysis or a new multiarm clinical trial within which each mucolytic agent (simethicone, dimethicone, pronase, and N-Acetylcysteine) was trialed individually and in various combinations against each other and with placebo.

We then examined whether combinations of mucolytic agents were superior to a single agent. Only the combination of simethicone and N-Acetylcysteine versus control was assessed in at least two studies, namely (Basford et al. 2016 and Monrroy et al. 2018), to facilitate a pairwise meta-analysis. Thus, a subgroup analysis was performed of these studies (Figure 6). This revealed a mean difference of −2.48 with a 95% confidence interval of −4.45 to −0.51. The Z statistics for the overall effect size was 2.47 and was statistically significant with a *P*-value of <0.01. The mean difference of −2.48 obtained when using simethicone in combination with N-Acetylcysteine was inferior to the mean difference obtained when using simethicone alone (−2.68). Thus, we can argue that there is no benefit in adding N-Acetylcysteine to simethicone as a preprocedure mucolytic agent. Having shown that mucolytic agents are superior to control in improving TMVS score and that simethicone alone may be superior, we then assessed what dose of simethicone was superior. This was assessed by conducting a subgroup analysis on the studies which used simethicone as a single premedication compared to control. These studies included Song et al. 2016 using 100 mg simethicone, Keeratichananont et al. 2010 using 133 mg of simethicone, and Monrroy et al. 2018 using 200 mg simethicone. The results were graphed on a forest plot (Figure 7).

As depicted, the mean difference for the 100 mg simethicone subgroup was −3.11, for the 133 mg simethicone subgroup was −4.22 and for the 200 mg of simethicone subgroup was −0.8. Thus, we can say that the most effective dose of simethicone in the included studies was 133 mg.

The timing of administration of the mucolytic agent prior to the OGD is an area identified within the systematic review of significant variation. Dosing timings ranged from 5 to 10 minutes up to 30 minutes before the commencement of the OGD. There is a concern that administering mucolytic agents as a preprocedure drink increases the risk of aspiration or aspiration pneumonia. We found no reports of aspiration or aspiration pneumonia in any of the 11,086 patients included in the 13 trials. To provide guidance on the most effective timing of administration of the mucolytic agent as a preprocedure drink, we conducted a subgroup analysis of the 6 studies included in the meta-analysis with studies subgrouped based on timing of administration of mucolytic agent. The subgroups were titled >20 minutes to denote the two studies whose timing was greater than 20 minutes (Keeratichananont et al. 2010 at 15–30 mins and Song et al. 2016 at 30 mins). The other subgroup was titled </ = 20 minutes for the 4 RCTs whose premedication was administered at less than or equal to 20 minutes (Asl et al. 2011 at 20 mins, Basford et al. 2016 at 5–10 mins, Monrroy et al. 2018 at 20 mins, and Zhang et al. 2018 at 20 mins).

The results were graphed on a forest plot (Figure 8) which revealed a mean difference for the >20 minutes group of −3.68. This was compared to the mean difference for the less than or equal to 20 mins group which was −2.69. Thus, the optimum timing of administration is likely greater than 20 minutes prior to the start of the OGD.

## 5. Conclusion

In conclusion, it is well established that GC is associated with significant morbidity and mortality if diagnosed late. The gold standard for diagnosis remains the OGD. OGD can miss subtle early lesions if these lesions are obscured by excess mucus. In this study, we have shown that mucolytic agents are effective in improving mucosal visibility by breaking up this mucus so that the stomach can physiologically remove it into the duodenum or the endoscopist can easily remove with flushes of water during the OGD. It appears reasonable to conclude that simethicone may be a suitable agent and that the addition of N-Acetylcysteine to simethicone may not be necessary. We have shown that an effective dose of simethicone is 133 mg and that it should be administered at least 20 minutes prior to the commencement of the OGD. A potential future avenue of investigation may now be to assess if the routine use of premedication with mucolytic agents is associated with increased lesion detection rate and thus improved survival rates and not just improved mucosal visualization, as this has not yet been demonstrated. Similarly, to date, no study has assessed the effect of improved mucosal visualization using mucolytic agents on the quality of imaged enhanced techniques of endoscopy and this certainly warrants further evaluation.

## Figures and Tables

**Table 1:** PICO model used to define search criteria for the search strategy to be used in the relevant bibliographic databases.

P	I	C	O
Population	Intervention	Comparison	Outcome
Adult patients undergoing elective nontherapeutic OGD	Premedication with a mucolytic agent	Placebo or another mucolytic agent	Improved mucosal visibility during OGD

**Table 2:** Summary of all studies included in the systematic review. Sim = Simethicone, NAC = N-Acetylcysteine, DMPS = Dimethicone, TMVS = Total mucosal Visibility Score, F = Female, M = Male.

Study ID	Methods	Participants	Interventions	Outcome measure	Anatomical site assessed	Timing of premedication	Result
Kuo et al. [8]	Double blinded randomized trial	160 patients randomized, F (92) M (68) mean age 50	5 intervention groups: (100 mg DMPS), (100 mg DMPS + 100 ml water), (2000 units of pronase + 100 ml water), (2000 units pronase + 1.2 g sodium bicarbonate + water 100 ml), (2000U pronase + 1.2 g sodium bicarbonate + 100 mg DMPS + 100 ml water) 0 control group	1 to 4-point scale	(4) gastric antrum, upper body, lower body, fundus	10 mins	No adverse effects reported. Premedication with pronase and DMPS in 100 ml water provides optimal visibility in upper gastrointestinal endoscopy.
Chang et al. [19]	An endoscopist-blinded, prospective, randomized study	147 randomized, F (77) M (70) mean age 46	4 intervention groups: (100 mg DMPS), (100 mg DMPS + 100 ml water), (20,000 IU pronase + 1.2 g Sodium bicarbonate + 100 mg DMPS + 100 ml water), (400 mg NAC + 100 mg DMPS + 100 ml water) 0 control group	1 to 4-point scale	(4) gastric antrum, upper body, lower body, fundus	20 mins	No adverse effects reported. Premedication with 20000 U pronase, 1.2 g of sodium bicarbonate, 100 mg of DMPS plus up to 100 mL of warm water provided best TMVS
Keeratichananont et al. [21]	Double blind, randomized, placebo controlled	121 randomized, F (66) M (55) mean age 57	1 intervention group: (133 mg sim + 60 ml water) 1 control group (60 ml water)	1 to 4-point scale	(6) oesophagus, gastric fundus, body, antrum, incisura, duodenum	15–30 mins	No adverse effects reported. Simethicone solution was more effective than placebo in reducing obscuring foam and bubbles at all areas of upper gastrointestinal tract.
Asl et al. [18]	Double blind, randomized, placebo controlled study	148 randomized, F (71) M (77) mean age 42	3 intervention groups: (100 mg DMPS + 100 ml water), (600 mg NAC+ 100 ml water), (100 mg DMPS + 600 mg NAC + 100 ml water)1 control group: (100 ml water)	1 to 4-point scale	(4) gastric antrum, upper body, lower body, fundus	20 mins	No adverse effects reported. 100 mg activated dimethicone in water up to 100 mL provided superior TMVS
Cha et al. [20]	Double blind randomized controlled trial	55 patients randomized, F (33) M (22) mean age 55	2 intervention groups: (20,000U pronase + 1g sodium bicarbonate + water), (80 mg sim)	1 to 3-point scale	(4) gastric antrum, upper body, lower body, fundus	10 mins	No adverse effects reported. Pronase solution provided a superior TMVS compared to simethicone.
Chang et al. [17]	Randomized, investigator-blinded study	1849 randomized, F (864) M (985) mean age 54	3 intervention groups: (5 ml water + 100 mg sim), (100 ml water + 100 mg sim), (100 ML water + 100 mg sim + 200 mg NAC) 0 control group	1 to 3-point scale	(7) oesophagus, fundus, upper body, lower body, antrum, duodenal bulb, D2)	10–30 mins	No adverse effects reported. 100 mg of simethicone improves TMVS, enhanced by addition of NAC
Kim et al. [22]	Multicentre, prospective, randomized, double blind study	143 randomized, F (56) M (87) mean age 59	2 intervention groups: (80 mg sim + 1g sodium bicarbonate + 20,000 U pronase + 100 ml water), (80 mg sim in 20 ml water) 0 control group.	1 to 4-point scale	(4) gastric antrum, upper body, lower body, fundus	10 mins	No adverse effects reported. Pronase + simethicone group provided superior TMVS than simethicone alone.
Basford et al. [15]	Randomized controlled clinical trial	126 randomized, F (64) M (62) mean age 62	2 intervention group: (50 ml water, 1G NAC, 60 mg sim), (50 ml water) 1 control group: (No pre-procedure drink)	1 to 4-point scale	(4) oesophagus, gastric body, fundus, antrum	5–10 mins	No adverse effects reported. Simethicone + NAC premedication significantly improves TMVS.
Song et al. [14]	Randomized, placebo controlled, endoscopist- blinded study	54 randomized, F (30) M (24) mean age 55	1 intervention group (100 mg sim + water) 1 control group: 5 ml water	1 to 4-point scale	(4) oesophagus, antrum and angularis of the stomach, body and fundus, duodenum	30 mins	No adverse effects reported. Simethicone solution provided significantly improved TMVS compared to placebo.
Elvas et al. [16]	Single-centre, prospective, double blind, placebo- controlled, randomized trial	300 randomized, F (148) M (152) mean age 62	2 intervention groups: (100 ml water + 100 mg sim), (100 ml water, 100 mg sim, 600 mg NAC) 1 control group (100 ml water)	Dichotomous: Excellent/Not excellent	(3) oesophagus, stomach body and antrum, duodenum	15–30 mins	No adverse effects reported. Simethicone solution improves mucosal visibility, inconclusive effect of addition of NAC.
Liu et al. [23]	Randomized, double blind, placebo controlled trial	7143 randomized, F (4159) M (2984) mean age 53	3 intervention groups: (20,000U pronase + 1g Sodium bicarb + 100 ml water), (80 mg simethicone + 100 ml water), (20,000U pronase + 80 mg simethicone + 1g Sodium bicarbonate + 100 ml water) 1 control group: (100 ml water)	1 to 3 scale	(5) oesophagus, cardia, fundus, body, antrum	20 mins	No adverse effects reported. Simethicone solution was superior to pronase solution in improving TMVS, however the combination was superior still.
Monrroy et al. [12]	Parallel assignment, double blinded, placebo- controlled randomized controlled trial	230 randomized F (156) M (74) mean age 49	4 intervention groups: (50 ml water), (W + Sim200 mg), (W + sim + NAC 500 MG), (W + sim + NAC 1000 MG) 1 control group: No intervention	1 to 4-point scale	(4) gastric antrum, upper body, lower body, fundus	20 mins	No adverse effects reported. Simethicone and NAC solution improves TMVS.
Zhang et al. [13]	Prospective, single blinded, randomized controlled trial	610 randomized, F (362) M (248) mean age 50	1 intervention group: (80 mg sim + 1G sodium bicarbonate + 20,000IU pronase) 1 control group: 10 mL lidocaine hydrochloride mucilage	1 to 4-point scale	(5) oesophagus, gastric antrum, upper body, lower body, fundus	20 mins	No adverse effects reported. Simethicone and pronase solution improved TMVS.

## List of Abbreviations

GC: Gastric cancer.
OGD: Oesophagogastroduodenoscopy
TMVS: Total mucosal visibility score.
